# Early Highly Pathogenic Porcine Reproductive and Respiratory Syndrome Virus Infection Induces Necroptosis in Immune Cells of Peripheral Lymphoid Organs

**DOI:** 10.3390/v17030290

**Published:** 2025-02-20

**Authors:** Jiawei Xu, Caiyun Huo, Yaling Yang, Jun Han, Lei Zhou, Yanxin Hu, Hanchun Yang

**Affiliations:** Key Laboratory of Animal Epidemiology of Ministry of Agriculture and Rural Affairs, National Key Laboratory of Veterinary Public Health and Safety, College of Veterinary Medicine, China Agricultural University, No. 2 Yuanmingyuan West Road, Beijing 100193, China; cauxjw@cau.edu.cn (J.X.); huocaiyun@cau.edu.cn (C.H.); yangshiyin284@gmail.com (Y.Y.); hanx0158@cau.edu.cn (J.H.); leosj@cau.edu.cn (L.Z.)

**Keywords:** highly pathogenic porcine reproductive and respiratory syndrome virus (HP-PRRSV), peripheral lymphoid organs, immune cell, necroptosis

## Abstract

The highly pathogenic porcine reproductive and respiratory syndrome virus (HP-PRRSV) has caused huge economic losses to the pig industry in China. This study evaluated the damage to peripheral immune tissues in the early infection of HP-PRRSV, including the hilar lymph nodes, mandibulares lymph nodes, inguinales superficials lymph nodes, spleens, and tonsils. HP-PRRSV infection led to a reduction in CD4^+^ and CD8^+^ T cells, as well as CD19^+^ B cells, in the tonsils. Additionally, CD163^+^ macrophages and CD56^+^ NK cells increased in all peripheral lymphoid organs, with NK cells migrating toward the lymphoid follicles. However, no significant changes were observed in CD11c^+^ dendritic cells. RNA-seq analysis showed the down-regulation of T and B cell functions, while macrophage and NK cell functions were enhanced. Gene Ontology (GO) and KEGG pathway analysis indicated the up-regulation of necroptosis processes. Western blotting and immunofluorescence confirmed that HP-PRRSV induced PKR-mediated necroptosis in immunocytes. This study provides new insights into the effects of early HP-PRRSV infection on peripheral immune organs, highlighting dynamic shifts in immune cell populations, virus-induced immunosuppression, and the role of PKR-mediated necroptosis. These findings improve our understanding of the immunomodulation induced by PRRSV infection.

## 1. Introduction

Porcine reproductive and respiratory syndrome (PRRS) is a serious disease that threatens the pig breeding industry [1]. PRRSV is a single-stranded, positive-sense RNA virus that belongs to the Arteriviridae family, Nidovirales order. Two genotypes of PRRSV have been identified including type 1 (EU) and type 2 (US), whereas strain variants often appear with the rapid evolution. PRRSV-1 is represented by the LV strain and PRRSV-2 by the VR-2332 strain [2,3]. PRRSV can infect the pigs of all ages, and reproductive failure, respiratory disease in young pigs, and reduced growth are the most common clinical signs following PRRSV infection [4,5]. Highly pathogenic PRRSV (HP-PRRSV) strains result in more serious clinical symptoms than low-pathogenic PRRSV (LP-PRRSV) [6]. Due to the antigenic diversity and the extensive genetic variations, PRRSV vaccines only offer partial cross-protection against PRRSV infection [7]. Therefore, the understanding of the pathogenesis mechanisms of PRRSV is crucial for exploring the effective preventive and therapeutic countermeasures.

PRRSV can proliferate in a variety of lymphoid organs [8], severely inhibiting the immune systems of animals, arousing infection, and accelerating the death of its hosts. A previous study has demonstrated that there is a significant reduction in CD4^+^CD8^+^ double-positive T cells in the peripheral blood at 3 days post infection (dpi), and further studies by other researchers found significant reductions in monocytes, B cells, and T cells in peripheral blood at 7 dpi [9,10]. HP-PRRSV also damages immunocytes in tissues, and studies have found a decrease in the number of CD163^+^ macrophages in the lung at 3 dpi and a decrease in T and B cells in lymph node and spleen at 7 dpi [11,12]. PRRSV infection also leads to a transient increase in NK cells in the peripheral blood, spleen, lymph nodes, and tonsils, which indicates that PRRSV can affect the status of immunocytes in the host after infection [13]. It has been noted that there are differences in the number of differentiated B cells and the rate of immune response in the lymph nodes and spleen following influenza virus infection [14], and T cell regulation in peripheral blood and secondary lymphoid organs may follow distinct rules [15]. Perhaps, there are also discrepancies in the response and status of immunocyte subsets in different peripheral lymphoid organs in the HP-PRRSV infection. Although there have been many studies on the changes in the number of immunocytes, few have also focused on the changes in their distribution after viral infection. To date, no study has comprehensively analyzed the numerical and distribution changes in multiple immunocytes in various types of peripheral lymphoid organs during the early stages of HP-PRRSV infection.

Necroptosis is a form of programmed cell death that differs from apoptosis and traditional necrosis, which is a hot topic in the field of cell research [16]. One pathway that initiates necroptosis is through a signaling cascade involving protein kinase R (PKR), receptor-interacting serine/threonine-protein kinase 3 (RIPK3), and mixed lineage kinase domain-like protein (MLKL) without the existence of caspase 8 [17,18,19,20]. The sequential phosphorylation of RIPK3 and MLKL conduce to the classical necroptotic pathway [21]. Existing research has shown that the cell death process in the lung of PRRSV-infected hosts varies at different times of infection. During the first week of infection, the lungs of infected pigs showed necroptosis or pyroptosis, while exogenous apoptosis became the mainstream regulatory death mode after the second week of infection [22,23]. Thus, necroptosis might be related to the damage to lymphoid organs that occurs in the early infection of HP-PRRSV. A large number of macrophage necrosis can be observed in the medulla area of PRRSV-infected thymus, and apoptotic positive signals are found in the tonsils, spleens, and ILNs of the infected pigs [23,24]. Furthermore, pyroptosis-positive signals are also observed in the ILNs of pigs infected with HP-PRRSV [25]. Therefore, the PRRSV infection can affect the programmed necrosis of cells in the immune organs, which shows a close relationship between programmed necrosis and immune system damage. However, the research on the mechanism of necroptosis in the peripheral lymphoid organs during the early infection of HP-PRRSV is still limited and needs to be further explored.

Herein, the study comprehensively investigated the pathological changes and immunocyte populations as well as the distribution in the peripheral lymphoid organs, including the hilar lymph nodes (HLNs), the mandibulares lymph nodes (MLNs), the inguinales superficials lymph nodes (ILNs), the spleens, and the tonsils at 3 dpi. RNA-seq was employed to elucidate the mechanisms of damage in the peripheral lymphoid tissues. Moreover, necroptosis of a variety of immunocytes induced by HP-PRRSV infection for 3 days in peripheral lymphoid organs were detected by Western blot and immunofluorescence. The findings contribute to elucidating the potential pathogenic mechanisms underlying lymphoid organ damage during the early stages of HP-PRRSV infection.

## 2. Materials and Methods

### 2.1. Ethics Statement

The animal experiments conducted in this study were carried out in accordance with the Chinese Regulations of Laboratory Animals. The study was undertaken in accordance with the Guidelines for the Care of Laboratory Animals (Ministry of Science and Technology of the People’s Republic of China, Beijing, China) and the Laboratory Animal Requirements of Environment and Housing Facilities (National Laboratory Animal Standardization Technical Committee). The protocol for the animal experiment was approved by the Laboratory Animal Ethical Committee of CAU, with approval no. AW70105202-2-02.

### 2.2. Viral Strains

The HP-PRRSV strain JXwn06 (GenBank accession number: EF641008) was propagated in MARC-145 cells in Dulbecco’s modified Eagle’s medium (DMEM, Gibco, Shanghai, China) with 10% fetal calf serum (Gibco) at 37 °C, and the virus titer was determined using the Reed–Muench method.

### 2.3. Antibodies

Anti-GAPDH (M20006), anti-phospho-MLKL (TA7420), anti-phospho-RIPK3 (TA4508), anti-RIPK3 (TD10141), anti-CD11c (TD7585), and anti-CD56 (PK22581) rabbit monoclonal antibody were purchased from Abmart (Shanghai, China). Anti-CD19 rabbit monoclonal antibody (DF7030) was purchased from Affinity Biosciences (Liyang, China). Anti-caspase-1 p20 and p10 (22915-1-AP), anti-CD3 (81324-1-RR), anti-MLKL (21066-1-AP), and anti-PKR (18244-1-AP) rabbit monoclonal antibody were purchased from Proteintech (Chicago, IL, USA). Anti-CD163 rabbit monoclonal antibody (93498) was purchased from Cell Signaling Technology (Boston, MA, USA), and anti-caspase-1 p20 rabbit monoclonal antibody (sc-398715) was purchased from Santa Cruz (Dallas, TX, USA). Anti-CD4 (4515-01) and anti-CD8α (4520-01) mouse monoclonal antibody were purchased from SouthernBiotech (Birmingham, AL, USA). FITC-labeled goat anti-rabbit IgG and Cy3-labeled goat anti-mouse IgG were purchased from Solarbio (Beijing, China).

### 2.4. Animals and Viral Challenge

Six-week old long-white pigs were purchased from the Beijing Center for SPF Swine Breeding and Management, which were free from the porcine reproductive and respiratory syndrome virus (PRRSV), African swine fever virus (ASFV), porcine circovirus type 2 (PCV2), and classical swine fever virus (CSFV) infection. The six pigs comprised two females and four males, and the specific information is displayed in Table S2. The pigs were randomly divided into two groups, and each group consisted of three pigs. The pigs of the virus-infected group were inoculated intranasally with 2 mL (1 × 10^5^ TCID_50_/mL) PRRSV strain of JXwn06, while the pigs of the control group were treated intranasally with 2 mL MARC-145 cell culture supernatant. At 3 dpi, pigs in two groups were euthanized. The clinical symptoms of experimental animals were observed daily until the end of the study for 3 days. The euthanasia procedure was carried out in accordance with the 2020 edition of the Guidelines for the Euthanasia of Animals. Zoletil was used for anesthesia, and the euthanasia was subsequently performed by exsanguination. The peripheral blood was collected from the experimental animals. The tonsils, spleen, HLNs, MLNs, and ILNs were collected to observe the gross lesions and histopathological changes.

### 2.5. PRRSV Detection of Serum by TCID_50_

To separate serum, the peripheral blood samples were collected into centrifuge tubes and centrifuged for 10 min at 3000 rpm. The MARC-145 cells were inoculated into the 96-well plates and cultured until the cells grew to 80% density; then, the gradient-diluted serum was added and incubated for 1 h at 37 °C. Subsequently, the serum was replaced with the DMEM medium containing 2% FBS. After the culture for 48 h, the cells were fixed with 4% paraformaldehyde and stained with the anti-PRRSV N protein antibody. After rinsing 3 times with PBS, the cells were incubated with a FITC-conjugated goat anti-mouse secondary antibody (Solarbio). The number of positive wells were observed using the fluorescence microscope (Olympus, Tokyo, Japan), and the fluorescence was observed by the picture capture system (OLYMPUS cellSens Standard 2.1). The Reed–Muench method was used to calculate TCID_50_/mL.

### 2.6. Histopathology

At necropsy, the tissue specimens were collected from the tonsils, spleens, HLNs, MLNs, and ILNs and fixed in 10% v/v buffered formalin. Formalin-fixed paraffin-embedded (FFPE) 4 μm sections were prepared on adhesion microscope slides for hematoxylin–eosin (H&E) staining. Histopathologic changes in organs were observed and scored under a bright microscope (Olympus). The pictures were taken by the picture capture system (ImageView, 4.11.22664). The degree of histopathologic lesions was assessed according to the scoring system described below: 0 = no microscopic lesions; 1 = extremely mild histopathologic changes; 2 = mild histopathologic changes; 3 = moderate histopathologic lesions; and 4 = severe histopathologic lesions. The specific scoring criteria of histopathologic lesions for each tissue are seen in Table S1.

### 2.7. Immunohistochemistry (IHC)

IHC was performed on FFPE sections of the tonsils, spleen, HLNs, MLNs, and ILNs. IHC FFPE 4 μm sections underwent deparaffinization, and antigen retrieval was performed. Microwave repaired the sections for 10 min, followed by cooling to room temperature. The slides were rinsed in PBS. Endogenous peroxidase activity was blocked by incubating sections with 3% hydrogen peroxide (H_2_O_2_) for 10 min. The slides were rinsed, incubated with PBS containing 10% normal goat serum for 30 min at room temperature (RT) to prevent unspecific background staining, and then incubated for 1 h and 30 min at room temperature with the primary antibody. Sections were subsequently rinsed with PBS and incubated with an anti-mouse or anti-rabbit biotinylated secondary antibody (Zhongshan Golden Bridge Biotechnology, Beijing, China) and labeled using an avidin–biotin–peroxidase procedure (Zhongshan Golden Bridge Biotechnology). The immunoreaction was visualized with a 3.3′-diaminobenzidine substrate (Zhongshan Golden Bridge Biotechnology). Sections were counterstained with hematoxylin, dehydrated in a graded alcohol series, and cover-slipped with the cover-slipping film (Sakura, Tokyo, Japan). Adequate positive (PPRSV-positive tissue) and negative controls (omission of primary antibody) were included in each immunolabelling assay. Distribution of positive signals in organs were observed under the light microscope (Olympus). The pictures were taken by the picture capture system (ImageView, 4.11.22664). Three fields were selected from each section to count the number of positive cells, and the means were calculated. In order to avoid the inclusion of false-positive cells, only the CD19-positive cells within lymphoid follicles were counted.

### 2.8. Quantitative Real-Time PCR (RT-qPCR)

The total RNA was extracted from the spleen, tonsil, HLN, MLN and ILN samples using TRIzol reagent according to the manufacturer’s protocol (Invitrogen, Waltham, MA, USA). And the complementary DNA (cDNA) was synthesized using a cDNA reverse transcription kit (Tiangen, Beijing, China) from 2 μg total RNA. The mRNA levels of the PRRSV ORF5 genes were relatively quantified with RT-qPCR using the TB Green^®^ Fast qPCR Mix (TAKARA Bio, Kusatsu, Shiga, Japan). The primers were designed using the Premier 5.0 software. The primers for the genes were as follows: ORF5 F: 5′-TTGTGCTTGCTAGGCCGC-3′; R: 5′-ACGACAAATGCGTGGTTATCA-3′; GAPDH: F: 5′-TCGGAGTGAACGGATTTG-3′; R: 5′-CCTGGAAGATGGTGATGG-3′; NLRP3 F: 5′-AGCCTTGAAGAGGAATGGATGG-3′; R: 5’-GCCTGGTGAAGCGTTTGTTGAG-3′. Each amplification reaction contained the 10 μL 2X TB Green Premix Ex Taq II Fast qPCR, 10 mmol/L forward primer, 10 mmol/L reverse primer, 2 μL cDNA template, and 6 μL DEPC water. The qPCR was performed on a 7500 Real-time PCR system (Applied Biosystems, Waltham, MA, USA). The cycling parameters used for RT-qPCR amplification were as follows: the holding stage at 95 °C for 30 s; 40 cycles of 95 °C for 5 s and 60 °C for 34 s; the melt curve stage at 95 °C for 15 s, 60 °C for 1 min, 95 °C for 30 s, and 60 °C for 15 s. The gene expression was determined using the 2^−ΔΔCt^ method, and GAPDH was used as an internal standard. Three technical replicates were set for each biological duplicate.

### 2.9. RNA Sequencing and Analysis

The total RNA was extracted from the spleen, tonsil, HLN, MLN, and ILN samples as mentioned above. RNA library construction and sequencing were performed by BGI (Beijing, China). The mRNA enrichment method was used to deal with the total RNA, and the constructed library was checked for quality and sequenced. The raw data were sequenced for quality control, and the resulting clean reads were aligned to the reference sequence. The research data used for the analysis have been uploaded to NCBI (BioProject ID: PRJNA1208406). The alignment rate and the distribution of reads on the reference sequence were used to determine whether the alignment results passed the second quality control. The sequencing data were filtered by the software SPAPnuke v2.3, and the filtered “clean reads” were saved in the FASTQ format. The clean reads were aligned to the Sus scrofa genome assembly with the software HISAT v2.0.4. The Bowtie2 v2.2.5 was used to map the clean reads to the reference gene sequence (transcriptome), and then, RSEM was used to calculate the gene expression level of each sample. Based on the principle of negative binomial distribution, the DEseq2 testing was performed using the method described in Michael I et al. [26]. Parameters: Q value (adjusted P value) <= 0.05. Venn analysis and KEGG enrichment analysis were performed by the results of differential gene detection.

### 2.10. Western Blotting

The total proteins were extracted from grinding fluid of tissues with RIPA lysis buffer (Beyotime, Shanghai, China) supplemented with 1 mM PMSF (Beyotime). Lysates were centrifuged, and the supernatant were collected. The protein concentration was detected with the BCA Protein Assay Kit (Solarbio). Proteins (20 μg) were separated via the 4% to 12% precast protein gels (GenScript, Piscataway, NJ, USA) and then were transferred to the nitrocellulose membranes. The membranes were blocked with the Western blocking buffer (Beyotime) for 2 h and incubated with the primary antibodies at 4 °C overnight. The membranes were washed with TBST buffer and incubated with the secondary antibodies for 1 h. Protein bands were visualized using Western Lightning Plus-ECL (Bio-rad, Hercules, CA, USA) and then analyzed with the software ImageJ (v1.53t).

### 2.11. Immunofluorescence

The tissue sections were dewaxed, rehydrated, blocked with the blocking buffer, and incubated with the mixture of the anti-caspase-1 antibody and the anti-CD163 antibody, anti-CD3 antibody, or anti-CD56 antibody overnight at 4 °C. Subsequently, the FITC anti-mouse secondary antibodies and the Cy3-coupled anti-rabbit secondary antibodies (Solarbio) were incubated successively for 30 min at 37 °C. The nuclei of the cells were counterstained with DAPI (Solarbio). The fluorescent signals were observed under the fluorescence microscope (Olympus). The fluorescent pictures were taken by the picture capture system (OLYMPUS cellSens Standard 2.1).

### 2.12. Statistical Analysis

Data were expressed as mean ± SEM. Two-way analysis of variance (ANOVA) was used for normality analysis, followed by Bonferroni statistical tests to compare differences between groups using GraphPad Prism 8.0. Statistical significance was defined as *p* < 0.05.

## 3. Results

### 3.1. HP-PRRSV Can Invade All Peripheral Lymphoid Organs During Early Infection

To ensure that the virus infection was in the early stage, the piglets were intranasally challenged with HP-PRRSV (2 × 10^5^ TCID_50_), and the clinical symptoms as well as the temperatures were detected. As shown in Figure S1A, the body temperature of HP-PRRSV-infected pigs was significantly increased compared with the control group at 3 dpi. This result suggested that 3 days after viral infection was the early stage of HP-PRRSV infection. In addition, the lungs of animals were observed during necropsy (Figure S1B). No significant clinical abnormalities were seen in the lungs in the control group. However, the lungs of the infected piglets displayed a pale coloration and rounded, blunt shape, which the lesions of white tiny spots could be seen at the periphery of the lobes. The results of the gross pathological changes suggested that HP-PRRSV could induce slight edema and interstitial pneumonic changes in the lung of piglets during the early infection. Viral titers in the peripheral blood serum were also determined using an immunofluorescence assay (Figure S1C). In comparison with the control group, the live viruses were detected in the serum of HP-PRRSV-infected group at 1 dpi, and the viral titers were dramatically increased with the duration of infection, especially at 3 dpi (Figure S1C). Thus, the HP-PRRSV infection can rapidly break through the respiratory barrier and enter the peripheral blood of infected animals at the early stage.

**Figure 1:**
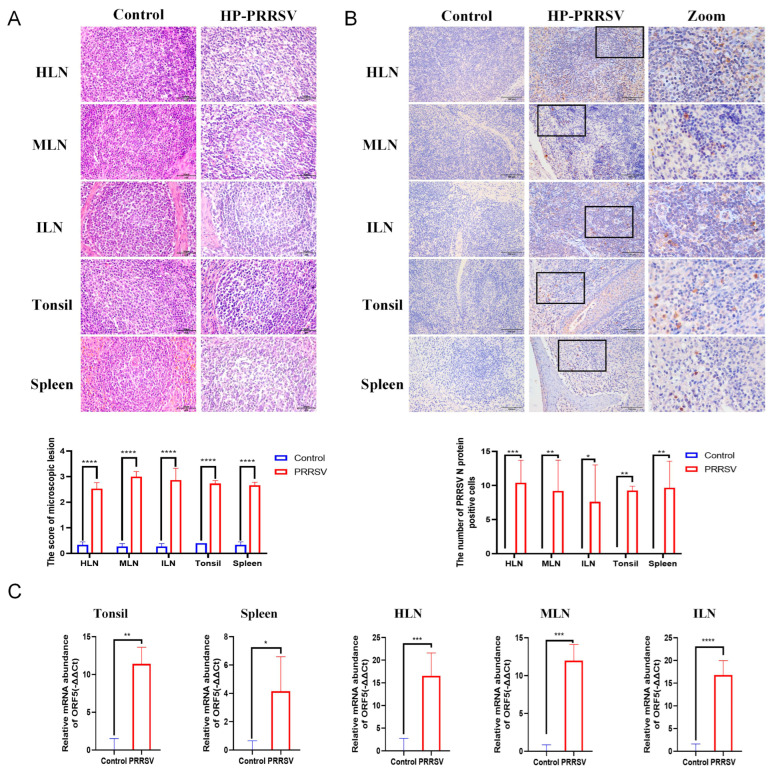
HP-PRRSV infects and damages the peripheral lymphoid tissues at the early stage. (**A**) The histopathological changes in the peripheral lymphoid organs at 3 dpi with H&E staining and the histopathological scores. ****, *p* < 0.0001. (**B**) IHC staining measured the counts and distributions of PRRSV N protein positive signals in peripheral lymphoid organs. *, *p* < 0.05; **, *p* < 0.01; ***, *p* < 0.001. (**C**) The relative mRNA abundance of ORF5 in peripheral lymphoid organs at 3 dpi with RT-qPCR; * *p* < 0.05; **, *p* < 0.01; ***, *p* < 0.001; ****, *p* < 0.0001. All pictures were taken under 40× objective.

As mentioned above, HP-PRRSV was in the early stage of infection in piglets at 3 dpi, and we further assessed the damages of peripheral lymphoid tissues in piglets after HP-PRRSV infection for 3 days. As seen in Figure 1A, no histopathological changes were observed in the peripheral lymphoid tissues of the non-infected piglets. Nevertheless, HP-PRRSV caused the severe histopathological changes in these tissues. The subcapsular and peritrabecular sinuses of the lymph nodes became widen, accompanied by the emergence of numerous gaps within both the medulla and cortex. Additionally, local hemorrhage occurred in the medulla of the HLNs. Gaps were also found in the tonsil lymphoid follicles and lamina propria as well as the white pulp of the spleen. The analysis of histopathological scores showed the significant difference between the control group and HP-PRRSV-infected group (*p* < 0.05). To clarify the location of the virus in the peripheral lymphoid organs during the early infection, IHC staining was performed as displayed in Figure 1B. Compared with the no positive signals in the peripheral lymphoid tissues of the control group, more PRRSV-positive signals were detected and mostly distributed in the medullary and marginal zones of the lymph nodes, the marginal zones of the tonsil, and the red pulp of the spleen. In addition, the positive signals were more clustered rather than scattered. Also, we quantified the HP-PRRSV in peripheral lymphoid tissues by RT-qPCR and found that the gene expression of PRRSV ORF5 was significantly increased in the infected group compared with the control group (*p* < 0.05). Based on the results of immunohistochemistry and RT-qPCR, the viral load in the spleen and tonsils was relatively lower than that in other peripheral lymphoid organs. Together, HP-PRRSV can invade the peripheral lymphoid organs in piglets at the early infection and proliferate and cause the tissue damages with a predominant depletion of intrinsic lymphocytes.

### 3.2. HP-PRRSV Infection Affects the Population of Immunocytes in the Peripheral Lymphoid Organs

**Figure 2:**
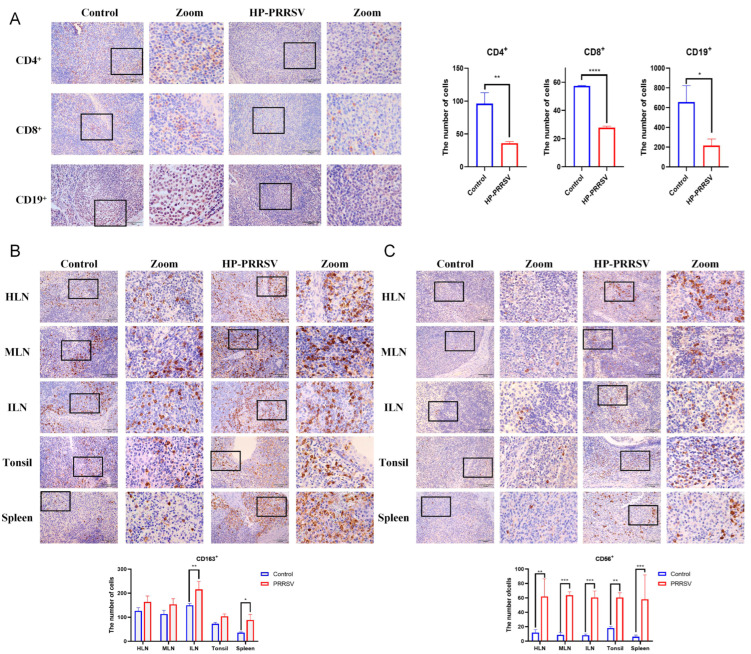
Effect of early HP-PRRSV infection on immunocytes in peripheral lymphoid organs. (**A**) Number and distribution of CD4^+^ T cells, CD8^+^ T cells, and CD19^+^ B cells in tonsils by immunohistochemistry. Number and distribution of CD163^+^ macrophages (**B**) and CD56^+^ NK cells (**C**) in peripheral lymphoid organs by immunohistochemistry. *, *p* < 0.05; **, *p* < 0.01; ***, *p* < 0.001; ****, *p* < 0.0001. All pictures were taken under 40× objective.

To clarify whether HP-PRRSV infection affected cell differentiation in peripheral lymphoid organs, we used the IHC techniques to semi-quantify the number of cells that were labeled with various different immune markers. As shown in Figure 2A, the majority of CD4^+^ and CD8^+^ T cells were distributed in the paracortex of lymph nodes, within lymphoid tissue situated in close to the crypt epithelium of the tonsils and in the vicinity of splenic lymphatic arteries in the control group. In addition, CD19^+^ B cells are predominantly located within the lymphoid follicles of peripheral lymphoid organs. In HP-PRRSV-infected piglets, the number of CD4^+^ T cells, CD8^+^ T cells, and CD19^+^ B cells was also significantly decreased in the tonsils, while no significant changes were observed in other peripheral lymphoid organs. The numbers of CD4^+^ T cells, CD8^+^ T cells, and CD19^+^ B cells in other peripheral lymphoid organs were listed in Figure S2A–C. CD8^+^ T cells were also decreased in the ILNs. Thus, the results demonstrate that the tonsils are exposed to HP-PRRSV earlier than other peripheral lymphoid organs, where the T cells and B cells are depleted at the early stage of HP-PRRSV infection.

CD163^+^ macrophages were predominantly distributed in the medullary and paracortical regions of lymph nodes, diffuse lymphoid tissues adjacent to the epithelium of the tonsil, and the red pulp of the spleen (Figure 2B). In HP-PRRSV-infected group, CD163^+^ macrophages were increased in all peripheral lymphoid organs, and significant differences appeared in the ILNs and spleen. NK cells in the control group were located in the medulla, limbic, and paracortical regions of the lymph nodes, the limbic region and lamina propria of the tonsils, and the periarterial lymphatic sheaths and red pulp of the spleen (Figure 2C). However, there was a significant increase in the number of CD56^+^ NK cells in each of the peripheral lymphoid organs after the virus attack. Interestingly, these NK cells were mainly recruited near the cortical area or lymphatic sheaths and tended to migrate toward the lymphoid follicles. Therefore, CD163^+^ macrophages and NK cells can proliferate significantly in the peripheral lymphoid organs during early HP-PRRSV infection.

CD11c^+^ dendritic cells are scattered and scarce in all peripheral lymphoid organs. Notably, our IHC semi-quantitative results showed no changes in the number and distribution of CD11c^+^ dendritic cells (Figure S2C).

### 3.3. RNA-Seq Analysis of Peripheral Lymphoid Tissues During Early HP-PRRSV Infection

**Figure 3:**
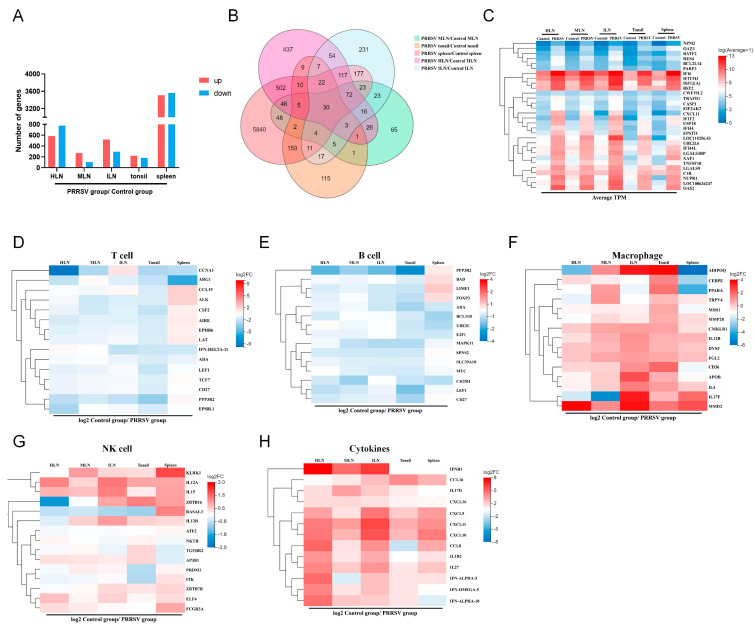
The early infection with HP-PRRSV has an impact on the DEGs of peripheral lymphoid tissues. (**A**) The number of DEGs between different groups with RNA-seq. (**B**) Venn plots showing the overlap of DEGs in peripheral lymphoid tissues. (**C**) The heatmap of expression of the 30 common DEGs in peripheral lymphoid tissues. (**D**) The heatmap of differential cluster of T cell-related genes. (**E**) The heatmap of differential cluster of B cell-related genes. (**F**) The heatmap of differential cluster of macrophage-related genes. (**G**) The heatmap of differential cluster of NK cell-related genes. (**H**) The heatmap of genes of cytokines.

To verify the effects of HP-PRRSV infection on the transcriptional levels of the peripheral lymphoid organs, total RNA was extracted from the peripheral lymphoid tissues of the HP-PRRSV-infected group and the control group, respectively, followed by RNA-seq analysis to detect the mRNA transcriptional profiles. As shown in Figure 3A, compared to the control group, there were 584 up-regulated differentially expressed genes (DEGs) and 773 down-regulated DEGs in the HP-PRRSV-infected HLNs, 267 up-regulated DEGs and 103 down-regulated DEGs in the MLNs, 517 up-regulated DEGs and 295 down-regulated DEGs in the ILNs, and 221 up-regulated DEGs and 180 down-regulated DEGs in the tonsils. The spleen had the highest number of DEGs, in which 3509 up-regulated genes and 3559 down-regulated genes were found. As the number of DEGs varied between different tissues, their commonalities were explored. A total of 8078 DEGs were identified in all tissues of the HP-PRRSV-infected and control groups, and 30 of these DEGs were common to all groups (Figure 3B,C). These DEGs, such as PKR (EIF2AK2) and caspase-1 (CASP1), were up-regulated in expression after HP-PRRSV infection. The results suggest that HP-PRRSV increases the number of DEGs in peripheral lymphoid tissues at the early stage of infection.

As described above, HP-PRRSV altered the number of immune cells exhibiting positivity for different surface markers within peripheral lymphoid organs. Here, the differential expressions of selected immune cell-related genes were also analyzed by RNA-seq analysis. Since there was no significant change in the number of CD11c^+^ dendritic cells in the peripheral lymphoid organs, we analyzed the expression of genes related to T cells, B cells, macrophages, and NK cells. As illustrated in Figure 3D,E, the majority of T cell-related genes and B cell-related genes exhibited decreased expressions in the peripheral lymphoid tissues. In contrast, as shown in Figure 2B,C, macrophage-related genes and NK cell-related genes showed a tendency of increase in the expression at the early stage of infection (Figure 3F,G), which was consistent with above histochemical results. During infection, immunocytes can recruit more immunocytes via the secretion of cytokines to eliminate the pathogens. We therefore analyzed the cytokine changes in the peripheral lymphoid tissues to investigate the relationship between varied subsets of immunocytes and cytokine secretion. As seen in Figure 3H, HP-PRRSV infection significantly elevated the expression levels of IL-4, IL-12A, IL-12B, and IL-15 cytokines in the peripheral lymphoid organs. In addition to the enriched cytokines that are described above, we found that the expression of several cytokines was significantly increased after HP-PRRSV infection. Analysis of changes in the expression of other cytokines in peripheral lymphoid organs showed that HP-PRRSV infection up-regulated the expression of several inflammatory factors and chemokines in peripheral lymphoid organs (Figure 3H). This suggests that HP-PRRSV infection is able to activate the macrophages and NK cells, causing a strong immune response.

**Figure 4:**
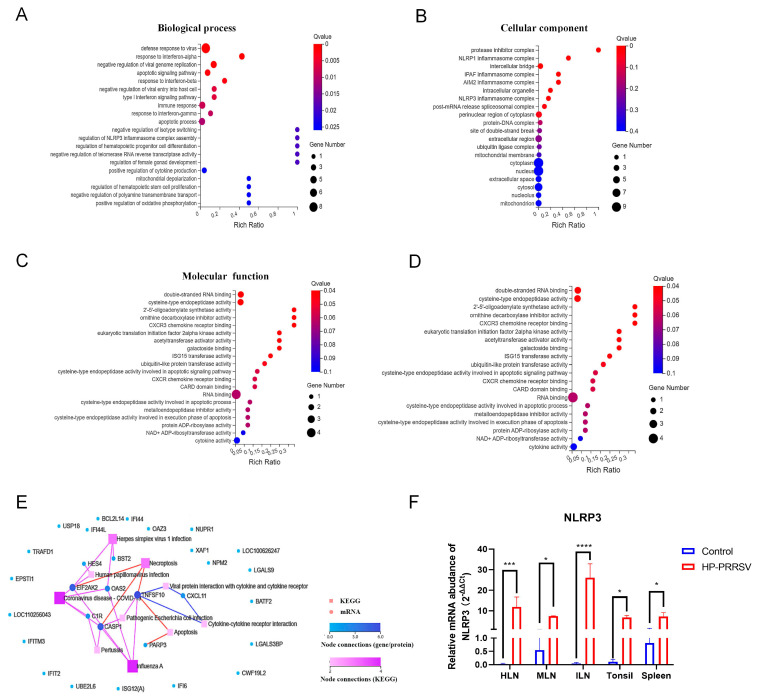
Early HP-PRRSV infection induces cellular necroptosis and promotes inflammatory factors’ production in peripheral lymphoid tissues. (**A**–**C**) Bubble diagrams obtained from GO analyses of common DEGs in terms of biological process, cellular component, and molecular function. (**D**) Bubble diagrams of KEGG analysis of common DEGs in peripheral lymphoid tissues. (**E**) KEGG network analysis chart of common DEGs in peripheral lymphoid tissues. (**F**) The relative mRNA abundance of NLRP3 in peripheral lymphoid organs at 3 dpi with RT-qPCR. *, *p* < 0.05; ***, *p* < 0.001; ****, *p* < 0.0001.

To further confirm the molecular phenotypic changes in the peripheral lymphoid organs at the early stage of HP-PRRSV infection, GO terms and KEGG pathway analysis on the above 30 common DEGs were further performed. GO terms included the biological process, cellular component, and molecular function. Biological process analysis results revealed terms such as response to interferon-alpha and interferon-beta, type I interferon signaling pathway, and regulation of NLRP3 inflammasome complex assembly (Figure 4A). Also, DEGs were significantly enriched in the terms of various cellular components including the NLRP1 and NLRP3 inflammasome complex in the HP-PRRSV-infected group (Figure 4B). The molecular functional analysis showed that DEGs were enriched in several terms such as CXCR3 chemokine receptor binding, ISG15 transferase activity, and CXCR chemokine receptor binding (Figure 4C). KEGG pathway analysis indicated that necroptosis, apoptosis, and viral–protein interactions with the cytokine and cytokine receptor pathways were associated with HP-PRRSV infection in the peripheral lymphoid organs (Figure 4D). KEGG pathway interaction maps were shown to further explore the connections among these 30 DEGs (Figure 4E), which found the enrichment of caspase-1, PKR, and TNFSF10 in the necroptosis pathway in the HP-PRRSV-infected group. RT-qPCR results showed a significant increase in the gene expression of NLRP3 in the peripheral lymphoid organs after HP-PRRSV infection (Figure 4F). Combined with GO and KEGG pathway analysis, the results demonstrated that the peripheral lymphoid organs underwent cellular necroptosis and NLRP3-related inflammatory responses at the early stage of HP-PRRSV infection.

### 3.4. HP-PRRSV Triggers PKR-Mediated Necroptosis of Immunocytes

**Figure 5:**
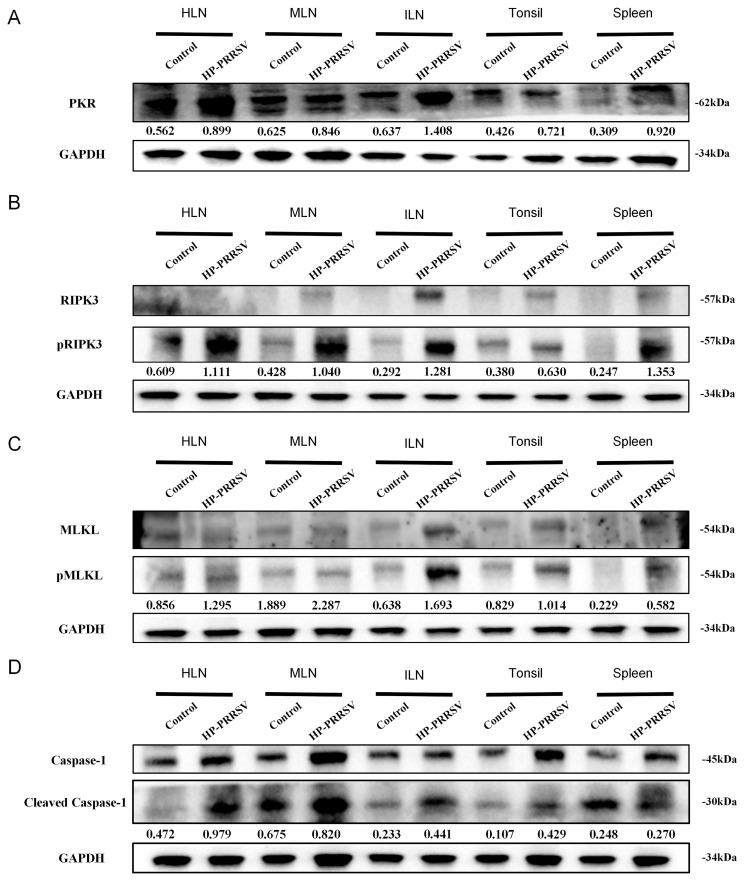
Early HP-PRRSV infection promotes cellular necroptosis and induces caspase-1 activation in peripheral lymphoid organs. Western blot detected expression levels of PKR (**A**), pRIPK3 (**B**), pMLKL, (**C**) and cleaved caspase-1 (**D**) in peripheral lymphoid organs.

In degenerative diseases and inflammatory environments, necroptosis often shoulders the role of killing pathogen-infected or damaged cells. PKR protein activation promotes RIPK3 protein activation, and thus, phosphorylated RIPK3 induces MLKL membrane translocation. Phosphorylated MLKL also induces caspase-1 autocleavage and promotes the release of inflammatory cytokines. In order to confirm whether necroptosis occurred in the cells of the peripheral lymphoid organs, WB was adapted to measure the expressions of necroptosis-related proteins including PKR, RIPK3, and MLKL. As shown in Figure 5A–C, an increase in the level of PKR protein was observed in various peripheral lymphoid organs after 3 days of HP-PRRSV infection, accompanied by a tendency for the rise in the phosphorylation levels of RIPK3 and MLKL. In addition, the protein expression of caspase-1 p10 fragment (cleaved caspase-1) was markedly elevated in all peripheral lymphoid organs in the HP-PRRSV-infected group (Figure 5D). Taken together, HP-PRRSV can induce the PKR-mediated necroptosis of cells in the peripheral lymphoid organs during the early infection.

**Figure 6:**
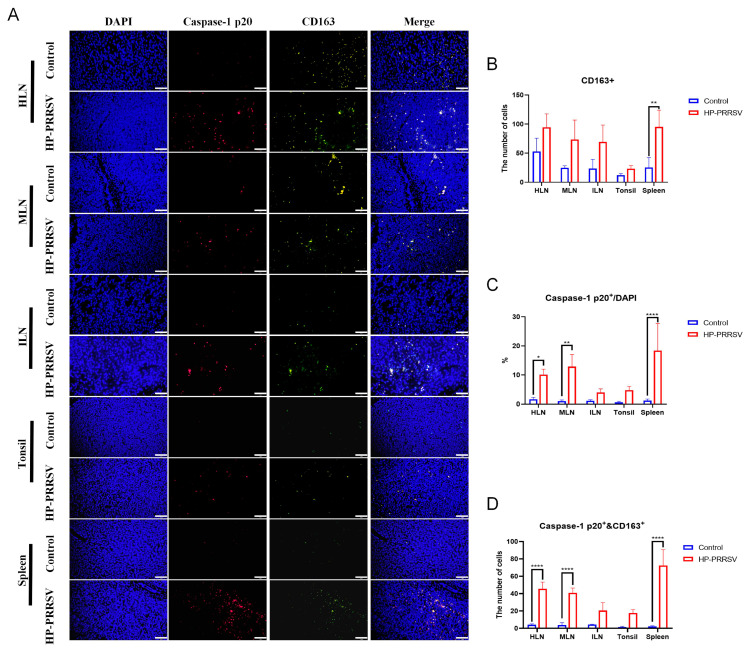
Early HP-PRRSV infection promotes the necroptosis in CD163^+^ macrophages. (**A**) Immunofluorescence detected the colocalization (in yellow) of caspase-1 p20 (in red) and CD163^+^ cells (in green) in peripheral lymphoid tissues. (**B**) Semi-quantity of CD163^+^ cells in peripheral lymphoid organs. The length of the scale bar was 50 μm. (**C**) The percentage of caspase-1 p20^+^ cells in the total number of cells observed in the field of views. (**D**) The number of CD163^+^ and caspase-1 p20^+^ double-positive cells in peripheral lymphoid organs. *, *p* < 0.05; **, *p* < 0.01; ****, *p* < 0.0001.

**Figure 7:**
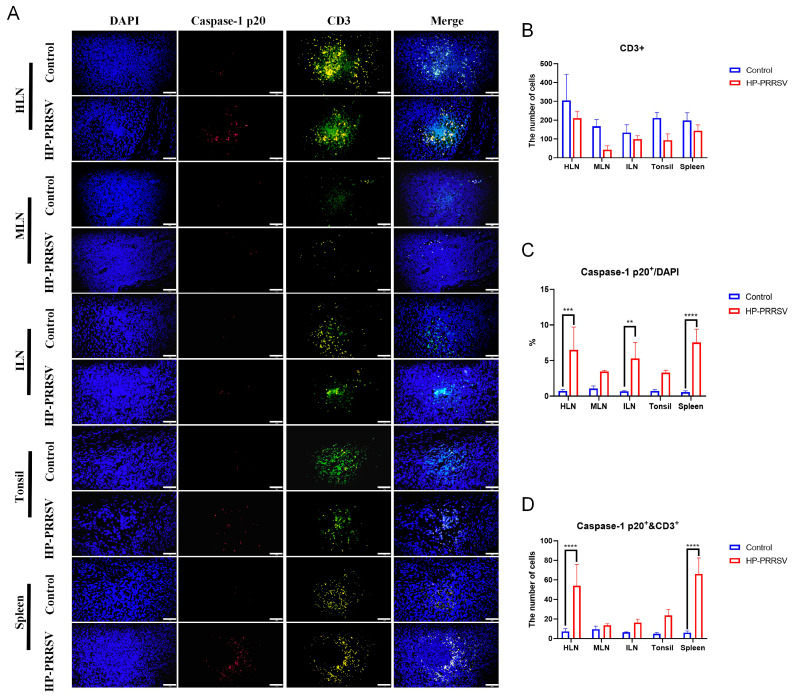
Early HP-PRRSV infection promotes the necroptosis in CD3^+^ T cells. (**A**) Immunofluorescence detected the colocalization (in yellow) of caspase-1 p20 (in red) and CD3^+^ cells (in green) in peripheral lymphoid tissues. The length of the scale bar was 50 μm. (**B**) Semi-quantity of CD3^+^ cells in peripheral lymphoid organs. (**C**) The percentage of caspase-1 p20^+^ cells in the total number of cells observed in the field of view. (**D**) The number of CD3^+^ and caspase-1 p20^+^ double-positive cells in peripheral lymphoid organs. **, *p* < 0.01; ***, *p* < 0.001; ****, *p* < 0.0001.

**Figure 8:**
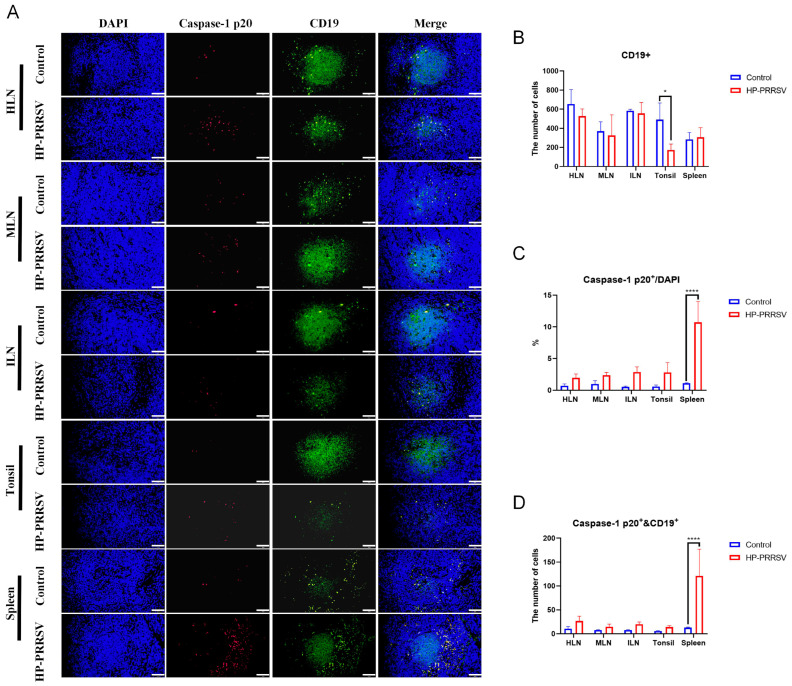
Early HP-PRRSV infection promotes the necroptosis in CD19^+^ B cells. (**A**) Immunofluorescence detected the colocalization (in yellow) of caspase-1 p20 (in red) and CD19^+^ cells (in green) in peripheral lymphoid tissues. The length of the scale bar was 50 μm. (**B**) Semi-quantity of CD19^+^ cells in peripheral lymphoid organs. (**C**) The percentage of caspase-1 p20^+^ cells in the total number of cells observed in the field of view. (**D**) The number of CD19^+^ and caspase-1 p20^+^ double-positive cells in peripheral lymphoid organs. *, *p* < 0.05; ****, *p* < 0.0001.

**Figure 9:**
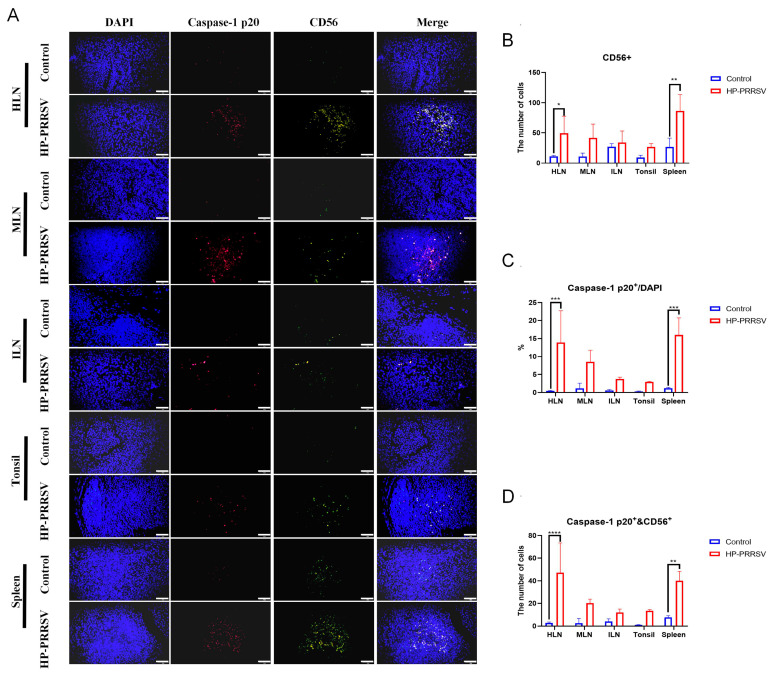
Early HP-PRRSV infection promotes the necroptosis in CD56^+^ NK cells. (**A**) Immunofluorescence detected the colocalization (in yellow) of caspase-1 p20 (in red) and CD56^+^ cells (in green) in peripheral lymphoid tissues. The length of the scale bar was 50 μm. (**B**) Semi-quantity of CD56^+^ cells in peripheral lymphoid organs. (**C**) The percentage of caspase-1 p20^+^ cells in the total number of cells observed in the field of view. (**D**) The number of CD56^+^ and caspase-1 p20^+^ double-positive cells in peripheral lymphoid organs. *, *p* < 0.05; **, *p* < 0.01; ***, *p* < 0.001; ****, *p* < 0.0001.

Subsequently, immunofluorescence was conducted to further validate the relationship between necroptosis and various immunocytes during the early infection of HP-PRRSV. As displayed in Figure 6A–C, CD163^+^ and caspase-1 p20 fluorescent signals were all significantly increased in the peripheral lymphoid organs of the HP-PRRSV-infected group, especially in the spleen, and this result was consistent with our findings obtained by immunohistochemistry and Western blot. Additionally, it was discerned that the majority of caspase-1 p20 fluorescent signals were situated within the same cells as those owning CD163^+^ signals. Semi-quantitative analysis showed that caspase-1 and CD163 double-positive signals were significantly increased in the infected group at the early infection when compared with the control group (Figure 6D). In terms of other immunocytes in the peripheral lymphoid organs, the colocalization of caspase-1 p20 and other immunocyte surface markers were also measured. In accordance with the IHC findings, the number of CD3^+^ T cells in peripheral lymphoid organs exhibited a decline following infection (Figure 7A,B). CD19^+^ B cells were significantly reduced only in the tonsils (Figure 8A,B), whereas the number of CD56^+^ NK cells showed an increase (Figure 9A,B). In addition, the ratios of caspase-1 p20 to total cells in the peripheral lymphoid organs were also elevated after HP-PRRSV infection (Figure 7C, Figure 8C and Figure 9C). For double-positive cells, an elevation in the quantity of CD3 and caspase-1 double-positive cells subsequent to HP-PRRSV infection was discerned mainly in the HLNs, tonsils, and spleen (Figure 7D). CD19 and caspase-1 p20 double-positive cells mainly in the spleen and CD56 and caspase-1 p20 double-positive cells mainly in the HLNs and spleen were observed to be increased following HP-PRRSV infection (Figure 8D and Figure 9D). Above all, HP-PRRSV could activate the PKR-mediated-necroptosis in the CD163^+^ macrophages, CD19^+^ B cells, CD56^+^ NK cells, and CD3^+^ T cells during the initial stages of infection.

## 4. Discussion

In this study, an early infection model of HP-PRRSV in vivo was established by euthanizing the animals at 3 days after transnasal tapping. We observed the gross pathological and histopathological changes in lymphoid organs in the early stage of HP-PRRSV infection, compared to control ones. It was clear that HP-PRRSV could cause damages to proliferate in the peripheral lymphoid organs and cause different degrees of damage. IHC results indicated that these viruses were mostly distributed in the medullary or cortical marginal zone. H&E staining illustrated that empty spaces were seen in the cortex and medulla of the lymph nodes, in the lymphoid follicles and lamina propria of the tonsils, and in the white pulp of the spleen. The gaps may result from the efferentation, migration, and even depletion of immunocytes. These results were consistent with the symptoms of lymph node edema noted in the literature by other investigators [27,28], suggesting that the virus was responsible for invading and damaging the lymph nodes. It has been shown that HP-PRRSV caused a decrease in T cell subsets in the thymus and a decrease in B cell subsets in the lymph nodes. The reduction in these cells is likely to be closely related to atrophy of the germinal centers and reduction in medullary cells in the thymus and lymph nodes [29]. Our observations of lesions were concentrated on the initial stage of HP-PRRSV infection. The findings indicated that the virus could cause damage to the immune system as early as 3 days post infection.

HP-PRRSV can suppress the host immune system through the programmed cell death. It has been shown that apoptotic signals can be detected in the lung tissues, lymphoid tissues, and bone marrow of piglets, which led to the depletion of immune cells [29]. The study has shown a decrease in the number of CD4^+^ and CD8^+^ T cells in the peripheral blood after HP-PRRSV infection, suggesting that HP-PRRSV infection leads to T cell depletion [30]. PRRSV interferes with the development of normal T cells in the thymus and impairs the induction of cytotoxic T cells and down-regulates T cell and NK cell markers [31,32,33]. There is also a decrease in the number of B cells in the lymph nodes and spleen after HP-PRRSV infection. In our study, gaps produced by immunocyte depletion were also displayed in the peripheral lymphoid tissues of piglets at the early stage of HP-PRRSV infection. In order to investigate whether the early HP-PRRSV infection affected the phenotypic expression of immunocytes, a series of IHC experiments were designed to examine the expressions of different immune antigens in the peripheral lymphoid tissues. A reduction in the quantity of CD4^+^ and CD8^+^ positive T cells was shown within the tonsils during the initial stage of HP-PRRSV infection, accompanied by a decline in the count of CD19^+^ B cells. Transcriptome analysis also indicated a decrease in the expressions of T cell-related and B cell-related genes, which echoed the decrease in CD4^+^ and CD8^+^ T cells revealed by the IHC results. Based on the results of the experiments, it is speculated that T cells can detect the viral invasion in the early infection and actively fight against the virus, with the tonsils being an important line of defense involved in this early immune response.

In contrast to the decrease in CD4^+^ T cells, CD8^+^ T cells, and CD19^+^ B cells, there was a significant increase in the number of CD163^+^ macrophages and CD56^+^ NK cells in the peripheral lymphoid organs upon the infection. Here, the transcriptome results demonstrated the elevated expressions of specific genes that were positively involved in the regulation of macrophages and NK cell activation, including NLRK1 and CMKLR1. Thus, macrophages and NK cells might be recruited to the peripheral lymphoid organs to facilitate the viral clearance during the initial stage of HP-PRRSV infection without depreciation or depletion. CD163 is known to be an important binding protein site for HP-PRRSV to invade cells. Previous studies have demonstrated that the CD163 gene can significantly control the HP-PRRSV infection if knocked down or removed [34,35,36]. Therefore, HP-PRRSV may replicate in the aggregated CD163^+^ macrophages, and this process may facilitate the HP-PRRSV to escape host immunity.

NK cells are classified into two distinct subsets, designated as CD56bright and CD56dim, based on the density of CD56 receptors on their cell surfaces [37]. The former NK cells mainly produce the high levels of cytokines, whereas the latter one exhibits a greater propensity for the cytotoxic activity [38]. In this study, CD56^+^ NK cells were found to be significantly increased in all peripheral lymphoid organs at the early stage of infection compared with the non-infected tissues. According to the transcriptome heatmaps, cytokines such as CCL8, IL-12, and IL-15 may play a role in recruiting the macrophages and NK cells. This suggests that CD56^+^ NK cells can also actively fight against the virus and might play a role in the secretion of the cytokines and the activation of the other immunocytes. Previous research has confirmed that NK cells are able to move towards the B cell follicles in response to the SIV infection, thereby controlling the spread of the virus [39]. In HP-PRRSV infection, we also observed that CD56^+^ NK cells migrated toward the cortical area or the lymphatic sheaths, with a tendency to encircle lymphoid follicles. It can be reasonably inferred that the recruitment and migration of NK cells to the cortical area represents an additional mechanism of immune defense adopted by the organism in response to the invasion of HP-PRRSV.

The medullary resident dendritic cells are demonstrated to migrate to the paracortical area during inflammation in the lymph nodes and leave gaps [40]. However, alterations in the CD11c^+^ dendritic cell population during the HP-PPRSV early infection were not noted in this study in the infected peripheral lymphoid tissues. This might be attributed to the lack of impact on the dendritic cells of the virus or the small number of dendritic cells present in the peripheral lymphoid organs as previously described [41], which made the discernible changes in their quantity difficult to discern.

Cytokines play a pivotal role in the host’s defense against viral diseases. However, they are also a double-edged sword, as the excessive immune response could stimulate a severe inflammatory response that would cause irreversible tissue damage. HP-PRRSV infection could lead to severe inflammatory lung damage [28]. Some studies have shown that HP-PRRSV infection differentially elevated cytokine levels such as IL-1β and IL-8 in peripheral blood and alveolar washings [42,43]. In this study, transcriptomics results showed that the expressions of several inflammatory cytokines and chemokines such as IL-12A and IL-4 were increased in the peripheral lymphoid organs during the early HP-PRRSV infection. GO analysis also showed multiple NLRP3-associated inflammatory responses upon infection. This finding suggests a correlation between inflammatory responses and damage to peripheral lymphatic organs in the early stage of HP-PRRSV infection.

Necroptosis is an important program of organismal defense and is recognized as a highly pro-inflammatory process because it leads to the release of various molecular patterns [44]. To date, more comprehensive studies have focused on the induction of host cellular apoptosis by HP-PRRSV, whereas fewer studies have been undertaken on the correlation between HP-PRRSV and necroptosis. In this study, RNA-seq analysis validated the occurrence of necroptosis in the infected piglets at the early stage, and the genes of PKR were enriched in the necroptosis pathway. By detecting the necroptosis-related proteins including PKR, pRIPK3, pMLKL, and cleaved caspase-1, it seemed that the early HP-PRRSV infection was able to induce the necroptosis of immunocytes in peripheral lymphoid organs by up-regulating the PKR expression, thereby activating the RIPK3-MLKL pathway. Furthermore, the results of immunofluorescence demonstrated that HP-PRRSV activated the necroptosis mainly in CD163^+^ macrophages, CD3^+^ T cells, CD19^+^ B cells, and CD56^+^ NK cells in the peripheral lymphoid organs during the early infection. While necroptosis plays a role in controlling viral dissemination and recruiting immune cells to defend against viral invasion, the pro-inflammatory factors secreted by necroptosis might also contribute to damage, as evidenced by symptoms such as edema and congestion in the peripheral lymphoid organs [45,46]. Here, the effects of necroptosis occurred in the CD163^+^ macrophages, CD3^+^ T cells, CD19^+^ B cells, and CD56^+^ NK cells on the HP-PRRSV infection are uncertain and worthy of further explored. The inhibition of necroptosis or the control of the inflammatory response might be therapeutic targets during the early treatment of HP-PRRSV.

## 5. Conclusions

In conclusion, the findings of this study indicate that early HP-PRRSV infection (3 dpi) results in the depletion or migration of immune cells in peripheral lymphoid organs, leading to the formation of gaps in the tissues. This process appears to be associated with necroptosis in immunocytes. Notably, the study reports for the first time a significant increase in and migration of CD56^+^ NK cells within these organs. Early HP-PRRSV infection also results in a marked reduction in CD4^+^ T cell, CD8^+^ T cell, and CD19^+^ populations in the tonsils, and a decrease in CD8^+^ T cell population in the ILNs, while CD163^+^ macrophage populations increase across all peripheral lymphoid organs. These findings provide new insights into the mechanisms of peripheral lymphoid organ damage during early HP-PRRSV infection and offer potential avenues for future research on the effects of PRRSV on immunocytes.

## Data Availability

Research data pertaining to this article can be found in the NCBI using BioProject ID: PRJNA1208406.

## Supplementary Materials

The following supporting information can be downloaded at: https://www.mdpi.com/article/10.3390/v17030290/s1, Table S1: Scoring descriptions of peripheral lymphatic organs; Table S2: Experimental animal information; Figure S1: HP-PRRSV infection can effectively attack experimental animals in 3 days; Figure S2: Some immunocytes subsets were unchanged at 3 days of HP-PRRSV infection.

## Abbreviations

The following abbreviations are used in this manuscript:

HP-PRRSV	highly pathogenic porcine reproductive and respiratory syndrome virus
HLN	hilar lymph node
MLN	mandibulares lymph node
ILN	inguinales superficials lymph node
dpi	days post infection
FFPE	formalin-fixed paraffin-embedded
IHC	immunohistochemistry
H&E	Hematoxylin–eosin
