# Supplementary material for: Early Highly Pathogenic Porcine Reproductive and Respiratory Syndrome Virus Infection Induces Necroptosis in Immune Cells of Peripheral Lymphoid Organs
Source: Viruses. 2025 Feb 20;17(3):290. doi: 10.3390/v17030290 (PMC11946179; doi:10.3390/v17030290)
Supplement: Supplementary file 1 [file viruses-17-00290-s001.zip › viruses-3462401-supplementary.pdf]

Supporting information

Jiawei Xu, Caiyun Huo, Yaling Yang, Jun Han, Lei Zhou, Yanxin Hu*, Hanchun Yang*

National Key Laboratory of Veterinary Public Health and Safety, Key Laboratory of Animal Epidemiology of Ministry of Agriculture and Rural Afairs, College of Veterinary Medicine, China Agricultural University, No. 2 Yuanmingyuan West Road, Beijing, 100193, China; cauxjw@cau.edu.cn (J.X.); huocaiyun@cau.edu.cn (C.H.); yangshiyin284@gmail.com (Y.Y.); hanx0158@cau.edu.cn (J.H.); leosj@cau.edu.cn (L.Z.)

***Correspondence:** yanghanchun1@cau.edu.cn; huyx@cau.edu.cn

Table S1. Scoring descriptions of peripheral lymphatic organs

Standard for evaluation of the lymph nodes	Score
No microscopic lesions.	0
The pathology was characterised by exceeding mild changes,when the presence of lymphocytes with slight necrosis, accompanied by eosinophilic infiltration within the paracortical area, was observed.	1
The pathology was characterised by slight changes, when a mild absence of lymphocytes was observed, accompanied by a small amount of plasma cell hyperplasia within the nodes and eosinophilic infiltration within the paracortical area.	2
The condition was classified as moderate,lymphocytopenia was evident within the nodes, accompanied with a small infiltrate of macrophages and eosinophils present within the paracortical area and hyperplasia of reticulocytes.	3
The pathology was characterised by severe changes when lymphocyte necrosis was reduced to small vacuoles, with lymphocyte necrosis and emptying in the lymph nodes, accompanied by macrophage infiltration, and a small macrophage infiltration in the paracortical area. Besides, reticulocyte hyperplasia was evident.	4
**Standard for evaluation of the tonsil**	**Score**
No microscopic lesions.	0
The pathology was characterised by exceeding mild changes,with a few eosinophilic infiltrated in diffuse tissue.	1
The pathology was characterised by slight changes, when a limited number of lymphocytes within the follicles were necrotic and diminished, and a modest number of epithelial cells were shed from the crypts.	2
The condition was classified as moderate,when the necrosis of lymphocytes was diminished, accompanied by a more pronounced cytopenia in lymphoid nodes, bruising of small blood vessels, and diffuse tissue with infiltration of small numbers of eosinophils and a few macrophages.	3
The pathology was characterised by severe changes with stasis of small blood vessels, shrinkage of lymph nodes, necrotic reduction of lymphocytes with cavities, epithelial cell detachment and a large number of degenerated and necrotic neutrophils and lymphocytes in crypts, and eosinophilic infiltration in diffuse tissue.	4
**Standard for evaluation of the spleen**	**Score**
No microscopic lesions.	0
The pathology was characterised by exceeding mild changes, including a small amount of lymphocyte necrosis and decreased numbers in the white marrow with a few eosinophilic infiltrated.	1
The pathology was characterised by slight changes, when the mild necrotic decrease of lymphocytes in the white marrow, accompanied by eosinophilic infiltration around the sheath arteries and gentle hyperplasia of the red marrow, was observed.	2
The condition was classified as moderate, with the following characteristics: red marrow hyperplasia, reduced lymphocyte necrosis in the white marrow, and the presence of small numbers of macrophages infiltrating the area surrounding the sheath arteries.	3
The pathology was characterised by severe changes with the hyperplasia of the red pulp, marked necrotic reduction of lymphocytes in the white pulp with a small macrophage infiltration, and necrotic reduction of lymphocytes in the form of small vacuoles around the splenic vesicles and sheath arteries.	4

Table S2. Experimental animal information

Animal Number	Group	Gender	Age
8	Control	female	6 week
9	Control	male	6 week
16	Control	male	6 week
3	HP-PRRSV infected	male	6 week
6	HP-PRRSV infected	male	6 week
12	HP-PRRSV infected	female	6 week

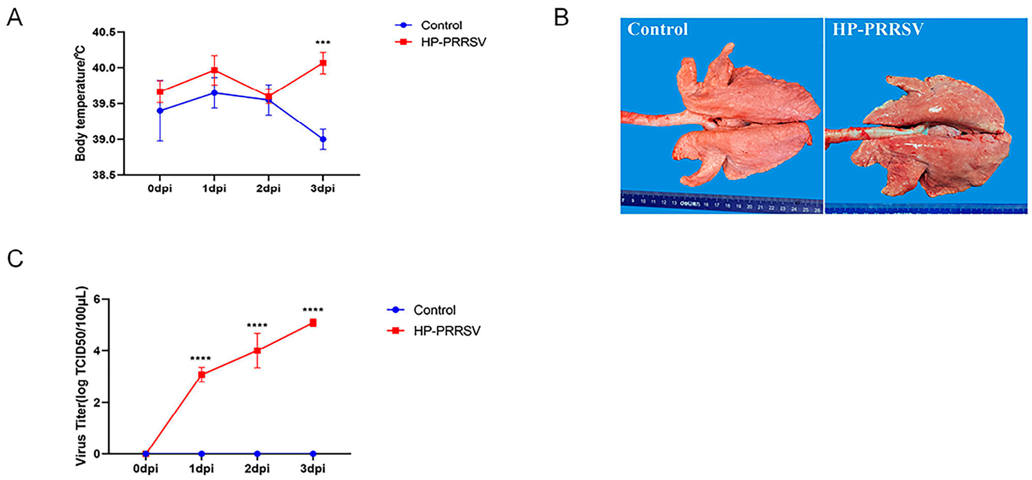

Figure S1 HP-PRRSV infection can effectively attack experimental animals in 3 days. (A) Changes in body temperature of HP-PRRSV infected animals (n=3). (B) Gross lesions in lung after exposure to HP-PRRSV for 3days. (C) Viral titers in peripheral blood serum of HP-PRRSV-infected animals were evaluated by $TCID_{50}$. ***, $P < 0.001$, ****, $P < 0.0001$.

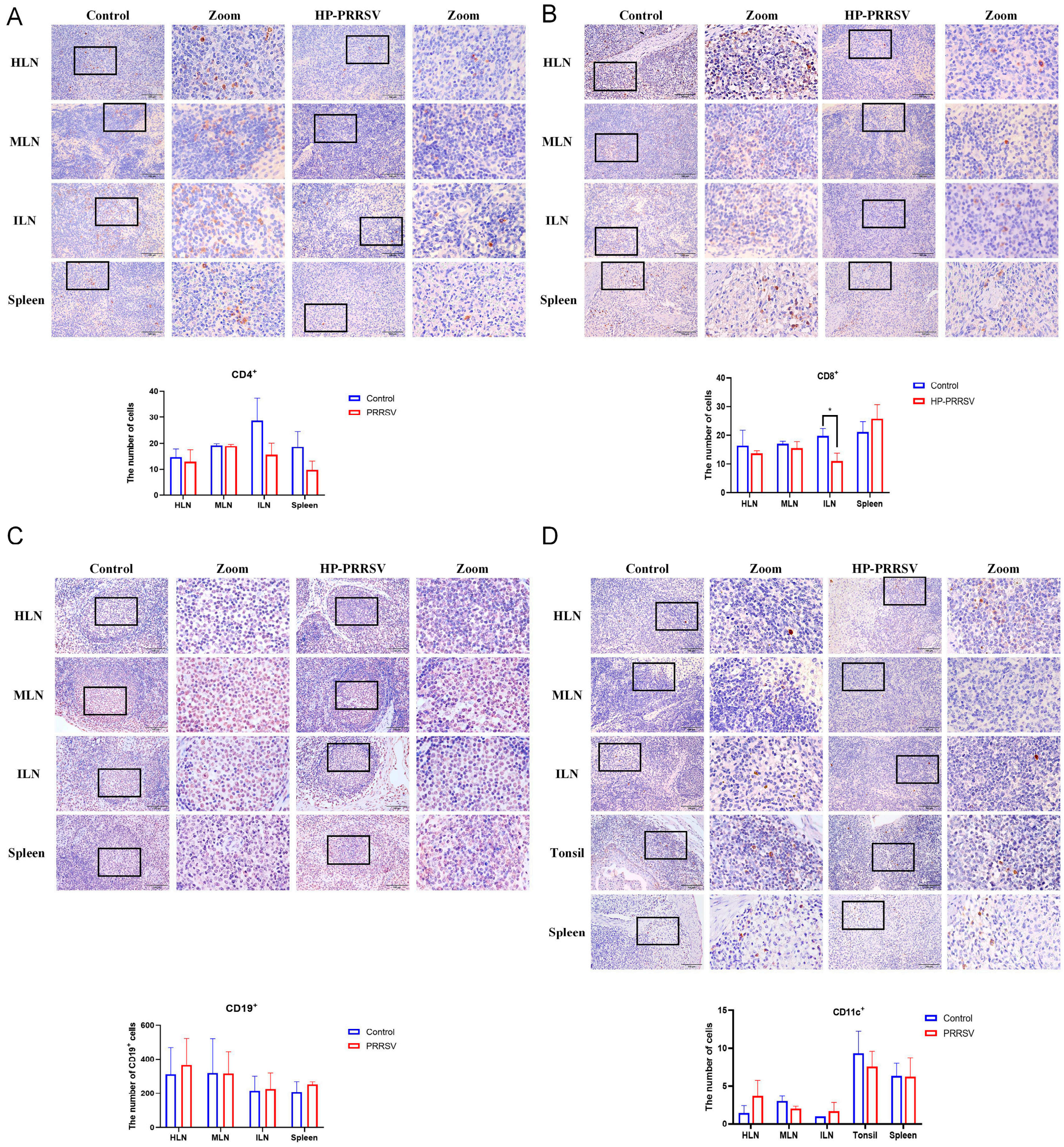

Figure S2 Some immunocytes subsets were unchanged at 3 days of HP-PRRSV infection. Number and distribution of $CD4^+$ T cells (A), $CD8^+$ T cells (B) and $CD19^+$ B cells (C) in the HLN, MLN, ILN and spleen by immunohistochemistry. (D) Number and distribution of $CD11c^+$ dendritic cells in peripheral lymphoid organs by immunohistochemistry. *, $P < 0.05$. All pictures were taken under 40× objective.
